# Development and validation of a comprehensive health literacy tool for adults in Hong Kong

**DOI:** 10.3389/fpubh.2022.1043197

**Published:** 2023-01-10

**Authors:** Cindy Yue Tian, Phoenix Kit-Han Mo, Dong Dong, Annie Wai-ling Cheung, Eliza Lai-Yi Wong

**Affiliations:** ^1^Faculty of Medicine, JC School of Public Health and Primary Care, The Chinese University of Hong Kong, Hong Kong, Hong Kong SAR, China; ^2^JC School of Public Health and Primary Care, Centre for Health Systems and Policy Research, The Chinese University of Hong Kong, Hong Kong, Hong Kong SAR, China

**Keywords:** health literacy scale, scale development, scale validation, Hong Kong Chinese adults, factor analysis

## Abstract

**Introduction:**

Health literacy (HL) refers to an individual's ability to process and use health information to make health-related decisions. However, previous HL scales did not fully cover all aspects of this concept. This study aimed to develop a comprehensive Hong Kong HL scale (HLS-HK) and evaluate its psychometric properties among Chinese adults.

**Methods:**

A scale of 31-item covering Nutbeam's framework, namely functional and interactive HL (FHL and IHL), and critical HL (CHL) within three subdomains: critical appraisal of information, understanding of social determinants of health, and actions to address social determinants of health, was developed based on previous literature review and Delphi survey. Cognitive interviews were performed to examine all items' face validity in terms of three aspects: comprehensiveness, clarity, and acceptability. A cross-sectional survey was conducted to investigate the scale's psychometric properties, including its internal consistency reliability, factorial structure validity, convergent validity, and predictive validity.

**Results:**

Nine interviewees participated in the cognitive interviews in October 2021. Based on the input from respondents, two items were deleted, two items were combined, and several items' wording was revised. The other items were clear and readable. Finally, 28 items remained. A total of 433 adults completed the questionnaire survey between December 2021 and February 2022. After excluding one item with low inter-item correlations, the scale's internal consistency reliability was acceptable, with a Cronbach's alpha of 0.89. Exploratory factor analysis produced a five-factor model, as shown in the original theoretical framework. These factors accounted for 53% of the total variance. Confirmatory factor analysis confirmed that the fit indices for this model were acceptable (comparative fit index = 0.91, root mean square error of approximation = 0.06, and root mean square residual = 0.06). The scale is also significantly correlated with theoretically selected variables, including education and self-rated health.

**Conclusion:**

The HLS-HK is a valid and reliable tool for evaluating HL. Compared with existing tools, this scale extended the operationalization of FHL, IHL, and CHL and fully operationalized the CHL *via* three subdomains. It can be used to understand the difficulties and barriers that people may encounter when they use health-related information and services.

## Introduction

Health literacy (HL) is crucial to empower individuals to make informed health decisions. It is usually described as one individual's ability to process and use health information to promote health (1). Previous studies suggested that people with limited HL skills tend to have poorer health outcomes (2–4), less utilization of preventive health services (5), higher hospitalizations (6), and healthcare costs (7, 8). One survey in eight European countries reported that at least 1 out of 10 participants had inadequate HL skills (9). There are challenges in embedding HL-related skills into effective disease prevention and health self-management. Assessing HL at a population level provides great potential to recognize populations most in need of support, deliver tailored interventions, and achieve better health outcomes in communities.

The measurement instrument is essential to understand people's HL levels. More than one hundred HL measurements have been developed during the last decades (10). The early HL measurement tools were criticized for their narrow focus on the capacity to read and understand written health information in a medical context (11–13). For example, the most commonly used HL tools, including the Test of Functional Health Literacy in Adults (TOFHLA) (11) and the Newest Vital Sign (NVS) (12), solely assess the reading ability of health-related materials through medical term recognition and numeracy test. Along with advancements in technology and the complex demands of health in modern society, scholars have realized that a broader set of competence is needed to access and use health-related information in everyday life. Align with this, several HL tools (14–17) expended to measure information seeking, communication skills, decision making, and critical thinking. However, recent systematic reviews highlighted that there is still no widely adopted measurement tool that could thoroughly reflect the current understanding of HL (18–22). Taking the lately dominant scales as examples, the Health Literacy Questionnaire (HLQ) (15) and the European Health Literacy Survey Questionnaire (HLS-EU) (23) did not include the skills necessary to address health concerns through civic engagement, which are essential HL skills to understand the social constructal cause of health-related issues and promote individual and community health. The ongoing evolution of HL measurement tools indicated the complexity of this concept and a demand for a comprehensive and reliable measure in this research field.

The construct underlying the measure is one of the most critical aspects of choosing and developing an appropriate measure. Nutbeam's framework of HL is widely cited as the conceptual basis and is seen by many HL researchers as useful in analyzing HL skills required in various contexts (24). This framework divides the primary skills of HL into three levels: functional health literacy (FHL), referring to individuals' basic literacy and numeracy skills for them to function effectively in their daily life; interactive health literacy (IHL), referring to individuals' cognitive and social skills to extract information from all kinds of forms of communication and to use this information for achieving better health outcomes; and critical health literacy (CHL), that is, individuals' higher level of cognitive and social skills which can be applied to critically analyze information and to use this information to gain better control over life events that affect health. The integrated model of HL proposed by Sørensen et al. is another vital framework in this research area. It described the competencies needed to access, understand, judge, and apply health information across the domains of health care, disease prevention, and health promotion (25).

Nutbeam's model was selected as the theoretical basis for the study because of its multifaceted understanding of CHL. Applying CHL has never been more needed than in these days when people have greater access to information and are expected to be actively engaged in healthcare. Compared with Sørensen's framework, Nutbeam emphasized that CHL includes the ability to question information and the awareness of the social determinants of health and the actions to modify these determinants (24). This emphasis is more explicitly linked to the latest understanding of CHL, that is, a range of abilities to read health-related information in a critical, active, and reflective manner to achieve an in-depth understanding of the world and explore political and social change in daily life (26–29). Taking the current pandemic of COVID-19 as an example, individuals need to know how to critically assess information when they are overwhelmed by the abundance of information, as well as how to make informed decisions to sacrifice some part of one's benefits for the sake of public good when they are asked to take certain interventions (e.g., vaccination and quarantine) to control infection. Therefore, CHL is needed to be seen in relation to critical consciousness and social responsibilities in promoting community health (27).

However, current HL measures using Nutbeam's framework and targeting adults failed to capture the full breadth of the ideas embedded in CHL. Previous scales (14, 30–35) mainly included the ability to question information quality as the component of CHL. For example, the 14-items HL scale (HLS-14) (30), which is widely cited in this research area, adopted items to measure subjects' ability to judge the quality of the information in terms of its reliability, validity, credibility, and applicability. Other competencies involved in CHL, namely realizing social and structural factors influencing health and taking social responsibilities for public health, were rarely addressed, and their operationalizations are still in progress. Chinn and colleagues made efforts to cover all aspects of CHL. But they faced the challenges of building items to thoroughly assess understandings of and ability to act on social determinants of health. They simply adopted three items involved in the capabilities for community empowerment and social engagement for health to reflect these abilities (14). The above revealed that the operationalization of CHL remains underexplored and more discussion is needed to explicitly link the theory and measurement in this domain.

Additionally, there is no rigorously validated HL scale for the general population in Hong Kong. Although several studies explored HL in the local community, the scales they used were either disease-specific or population-specific (36–40) or directly translated from existing ones without psychometric testing (41, 42). Hong Kong is facing the challenges posed by the increasing disease burden from chronic diseases and has a dual-track healthcare system encompassing public and private sectors. This system has been criticized for the long waiting times in public hospitals and high healthcare costs in private hospitals (43, 44). Under such circumstances, patients with chronic diseases are expected to actively engage in self-management, which requires adequate HL skills. With this respect, one reliable and valid HL scale will be useful to identify the attributes and barriers related to HL.

With all these in mind, our goal was to develop a new Chinese Hong Kong version of HL scale (HLS-HK) based on Nutbeam's framework and evaluate its psychometric properties among Chinese adults. Given that the theory and measurements of HL are still in the exploratory stage, our work would be feedback loops to improve the underlying theory of HL. We also expect that the scale would provide regional-level information related to health competency and facilitate more efforts to understand HL skills and their impact on health outcomes in Hong Kong.

## Methods

A four-stage research approach was adopted, including literature review, Delphi study, individual cognitive interview, and cross-sectional survey to develop and validate a comprehensive scale for HL. In the first stage, we conceptualized the framework of HLS-HK by conducting two scoping reviews (45, 46). In the next stage of the Delphi study (47), we deductively generated items based on the framework resulting from the first stage and invited stakeholders (i.e., healthcare providers and healthcare consumers) to assess the content validity of all draft items and provide additional items. The face validity accessed *via* individual cognitive interviews and psychometric analysis using a cross-section survey were reported in this paper.

### Cognitive interview

The cognitive interviews were conducted to test the face validity of the 31 items derived from previous literature reviews and the Delphi survey. In the interviews, interviewees were first asked to complete the scale and then were invited to give feedback on all items' comprehensiveness, clarity, and acceptability. Participants were recruited through a convenience sampling strategy. The eligibilities of participants were as follow: (a) permanent Hong Kong citizen; (b) aged 18 and above; (c) understand Cantonese. To keep the recruitment costs low, we sought the participation of individuals working or visiting our school to participate in the interview. To achieve a representative sample, we selected interviewees by considering a balance of gender, age, educational attainment, and income. The sample size for cognitive interviews was set between 5 and 15 subjects (48). Participant recruitment stopped when data saturation reached (48, 49).

### Cross-sectional validation survey

A psychometric evaluation was performed to examine the internal consistency, factorial structural validity, convergent validity, and predictive validity of the revised version of HLS-HK.

### Participants

Participants were recruited from registrants of an internet research service company Qualtrics to complete an online survey. Quota sampling was used to match the distribution of participants by gender, age group, and living district (i.e., New Territories, Kowloon, Hong Kong Island) to the results of the 2020 Hong Kong census (50). Facing resource constraints and challenges to reach the fixed quotas, the flexibility on all quotas was ±5%. The recommendation for a sample size used to scale validation should be at least 10 participants per item of the scale (51, 52). Therefore, in the present study, the sample size should be over 310 (=31^*^10). To ensure sufficient evidence for the reliability and validity of the newly developed scale and also consider the budget, the sample size of our survey was expected to be 400 participants.

### Measures

The questionnaire consisted of two parts: (1) health literacy assessment; (2) social demographic and health factors. It was built on Qualtrics survey software (version Dec 2021). All questions were designed to force response. We also set up one attention check question to maintain data quality by excluding respondents who were not paying close attention to survey questions.

#### Health literacy assessment

Participants' HL levels were measured using our newly developed scale HLS-HK and the 12-item short-form of the health literacy questionnaire (HL-SF12) (53). Regarding HLS-HK, items were rated on a 5-point Likert Scale, and the scores were summed. A sample item was “How often do you need help when you fill out medical forms?”. The item responses ranged from 1 = always to 5 = never. The HL-SF12 was developed based on Sørensen and colleagues' framework of HL (25). It is the short version of the HLS-EU (23) and has been validated in six Asia countries (54). Given the importance of Sørensen's framework in this research area, we used HL-SF12 as the reference scale. We contacted the authors of HL-SF12 and got their approval to use it in our study. In HL-SF12, the perceived difficulty of each health-related task was rated on 4-point Likert scales (ranging from 1 = very difficult to 4 = very easy). Example items included “On a scale from very easy to very difficult, how easy would you say it is to judge the advantages and disadvantages of different treatment options.”

#### Social demographic and health factors

The following social demographic characteristics were collected from the respondents: age, gender, district, education attainment, occupation, and monthly household income. Participants were also asked to self-report their health status and health behaviors. Health status was examined by participants' responses to the questions “In general, how would you rate your health” with five outcomes (1–5, ranging from “poor” to “excellent”). This question is often used to assess a person's overall wellbeing in terms of social, biological, and psychological health in epidemiological health field surveys (55, 56). Health behaviors were assessed by asking about participants' lifestyles related to smoking, physical activity, and alcohol use.

### Data analysis

Descriptive statistics were used to summarize study participants' demographic information and other health-related variables. Continuous variables were shown as mean and standard deviation (SD), and categorical variables were presented as numbers and frequencies. The reliability and validity analyses were performed to examine the psychometric properties of the HLS-HK. Data were analyzed using the SPSS (version 23) and R software (“psych” and “lavaan” package). The statistical significance level was set at a *p* < 0.05.

#### Reliability analysis

In reliability analysis, we focused on the internal consistency of the scale and subscales by testing Cronbach's alpha values. An alpha coefficient of 0.70 or greater is considered acceptable for reliability (57). Furthermore, corrected item-total correlations were tested. Two levels of inter-item correlations were suggested as acceptable: 0.2 (58, 59) and 0.3 (60, 61). We used the more liberal level of ≥0.2 in this study in order not to exclude some items on which some participants got high scores while others got low scores due to the heterogeneity within social demographic and health factors among subjects.

#### Validity analysis

We used exploratory factor analysis (EFA) and confirmatory factor analysis (CFA) to assess the construct validity of HLS-HK. The sample was randomly split into two independent subsets to undertake separate EFA and CFA analysis.

EFA was first performed to discover the common factor influencing a group of measured variables of HLS-HK. Before performing an EFA, we used Bartlett's Test of Sphericity and the Kaiser-Meyer-Olkin (KMO) test to evaluate whether the data was worth factor analysis. If the probability of Bartlett's Test of Sphericity is <0.05 or KMO is >0.7, we can move to the EFA (62). Next, EFA was performed using principal component analysis, oblimin rotation, and parallel analysis. The criterion for selecting the optimal number of factors is based on: eigenvalue >1 and scree test (57). It is recommended that the retain items have factor loading of 0.4 and above and without significant cross-loading onto other factors (i.e., those that load on over one factor were excluded) (57, 63, 64).

CFA was then performed using maximum likelihood estimation to validate the factor structure of the HLS-HK. In the present study, the following indices of model fitness were used: comparative fit index (CFI), root mean square error of approximation (RMSEA), and root mean square residual (SRMR). A CFI value ≥0.90 indicated an acceptable model fit; an RMSEA value ≤0.08 shows a good fit; and an SRMR value ≤0.08 can be considered a good fit, based on suggestions in previous literature (57, 65).

#### Bivariate analysis

We performed the bivariate analysis between the HLS-HK scores and HL-SF12 scores to examine the convergent validity using Pearson correlation coefficients. In the present study, HL-SF12 was considered as the reference. We expected the two scales produce similar results. Namely, a participant who got a high score of HLS-HK is likely to have a high score of HL-SF12. In addition, as both HLS-HK and HL-SF12 measured the main skills related to HL, all subscales from the two scales should be somewhat related.

Regarding predictive validity, we examine the relationship between HLS-HK and other theoretically selected variables (including age, education attainment, income, self-rated health status, and health behaviors) using the Mann-Whitney U test (two samples) or Kruskal-Wallis test (more than two samples). Given previous studies highlighted that people with low HL skills were likely to be older (66, 67), less educated (67, 68), with lower-income (68, 69), poorer self-rated health status (69, 70), and less healthy behaviors (70, 71), we hypothesized that HLS-HK levels were significantly correlated with the above variables.

### Ethical consideration

The Survey and Behavioral Research Ethics Committee of the Chinese University of Hong Kong accessed the protocol of this study and approved the study (Reference No. SBRE-20-793). The protocol for this study conformed to the principles embodied in the Declaration of Helsinki.

## Results

The results of the first two stages were published elsewhere (45–47) and briefly presented below. In the first stage, five content areas that we aimed to measure were identified, including FHL, IHL, and the following three subdomains of CHL: CHL-1: “critical appraisal of information” means an individual's ability to evaluate the quality of information; CHL-2: “understanding of social determinants of health” coveys an individual's understanding of the social structural factors that influence health outcomes; CHL-3: “actions to address social determinants of health” focuses on an individual's competency to translate knowledge into action to address the modifiable determinants of health (45, 46). In the second stage, the Delphi study was completed (47). We generated the first draft of the HLS-HK consisting of 34 items. A diverse panel of professionals (*n* = 12) and laypeople (*n* = 12) rated the relevance of all 34 items. The consensus, which was predefined as ≥70% of participants agreeing that the individual item is relevant in Round 3, was reached for 31 items with excellent content validity. This HLS-HK with 31 items was used to test its face validity in cognitive interviews.

### Cognitive interviews

Data saturation was achieved after nine interviews in October 2021. Table 1 presents the social demographic characteristics of the participants. Based on the input from interviewees, we deleted one item in FHL and one item in CHL-2, combined two items in CHL-3, and made a minor revision on several items' wording to make the scale concise. The other items were comprehensive, clear, and acceptable. Detailed results of cognitive interviews are presented in Appendix 1. Finally, a total of 28 items remained (Table 2). The revised version of HLS-HK within 28 items was adopted to test its psychometric properties in the following cross-sectional survey.

**Table 1:** Characteristics of interviewees.

**Subjects**	**Gender**	**Age group**	**Education**	**Income (HKD)**
1	Male	≥65	Primary and below	≥18,400
2	Female	≥65	Primary and below	<18,400
3	Female	45–64	Primary and below	<18,400
4	Male	18–44	Secondary and above	≥18,400
5	Female	18–44	Secondary and above	≥18,400
6	Female	18–44	Secondary and above	<18,400
7	Male	18–44	Secondary and above	<18,400
8	Female	≥ 65	Primary and below	<18,400
9	Female	≥ 65	Primary and below	<18,400

**Table 2:** Items resulting from cognitive interviews.

**Domain**	**Items**	**Corrected item-total correlations**	**Cronbach's alpha if item deleted**
FHL	How often do you^a^:		
	q1 …need help when you fill out medical forms	0.36	0.88
	q2 … find that characters cannot understand when you read instructions or leaflets from hospitals or clinics	0.50	0.88
	q3 …feel that the content is too difficult to understand when you read instructions or leaflets from hospitals or clinics	0.52	0.88
	q4 … have problems understanding health-related written information	0.55	0.88
IHL	How easy would you say it is to^b^:		
	q5 …find related information when you are ill and have questions on disease or health problems	0.59	0.88
	q6 … find related information when you are not ill but want to do something to further improve your health	0.59	0.88
	When you talk to a doctor, nurse, or pharmacist, how difficult would you say it is to^b^:	0.54	0.88
	q7 …give all the information they need	0.57	0.88
	q8 …ask the questions you want to ask	0.58	0.88
	q9 …ask further explain anything that you do not understand after they answer your questions	0.61	0.88
	q10 …extract the information you want	0.60	0.88
	q11 …understand the obtained information		
CHL-1	When you get information for health in daily life, how often do you consider the following^c^:		
	q12 …whether the information source is credible	0.46	0.88
	q13 …whether the information content is valid and reliable	0.45	0.88
	q14 …whether the publish time is appropriate	0.50	0.88
	q15 …whether other reliable sources support the facts or conclusions of this source	0.52	0.88
	q16 …whether the person or organization that produced the information have a bias	0.39	0.88
	q17 …whether the information is applicable to you	0.50	0.88
CHL-2	How do you agree about the following^d^:		
	q18 …socioeconomic status affects health	0.16^*^	0.89
	q19 …stress affects health	0.23	0.88
**Domain**	**HLS-HK items**	**Corrected** **item-total** **correlations**	**Cronbach's alpha** **if item deleted**
	q20 …being isolated from the community and workplace impacts health	0.31	0.88
	q21 …having little control over one's work impacts health	0.29	0.88
	q22 …poor childhood experience has an impact on one's physical/mental health when he or she becomes an adult	0.20	0.89
	q23 …good social relations contribute to health	0.31	0.88
CHL-3	How often do you^e^:		
	q24 …promote government to launch programmes about health promotion and disease prevention	0.28	0.88
	q25 …participate in community's or non-governmental organizations' initiatives in health promotion and disease prevention	0.24	0.89
	q26 …help your family members or a friend when they had questions concerning health issues	0.48	0.88
	q27 …seek information from others when you come up with questions concerning a health issue	0.37	0.88
	q28 …share and communicate your opinion about illness when you talk to a family member or friend	0.43	0.88

a Response options range from “1 = always” to “5 = never”.

b Response options range from “1 = very difficult” to “5 = very easy”.

c Response options range from “1 = never” to “5 = always”.

d Response options range from “1 = strongly disagree” to “5 = strongly agree”.

e Response options range from “1 = never” to “5 = always”.

* The corrected item-total correlations lower than 0.2.

### Cross-sectional validation survey

#### Social-demographic and health-related characteristics

The questionnaire survey was performed from December 2021 to February 2022. A total of 433 valid responses were collected after excluding those with data entry errors and speeders (i.e., respondents who completed the survey much more rapidly compared to others). In this study, we defined the cut-off point of speeders' completion time according to a soft launch of the survey (*n* = 40). In the soft launch, the median time to completion is 7.8 min, and we added half of the median completion time (i.e., 4 min) as the speeding check. The participants' social-demographic characteristics are displayed in Table 3.

**Table 3:** Characteristics of survey participants.

**Variables**	**Total (*****n*** = **433)**		**2020 Hong Kong census**
	***n***	**%**	**%**
**Gender**			
Male	217	50.1	44.9
Female	216	49.9	55.1
**Age groups**			
18–24	53	12.2	9.90
25–34	80	18.5	15.6
35–44	70	16.2	17.8
45–54	87	20.1	17.2
≥55	143	33.0	39.5
**District**			
HK island	78	18.0	16.6
Kowloon	140	32.3	30.6
New Territories	215	49.7	52.8
**Education attainment**			
Primary and below	10	2.3	
Secondary	118	27.3	
Post-secondary (non-degree course)	75	17.3	
Post-secondary (degree course)	230	53.1	
**Employment status**			
Full-time	315	72.7	
Part-time	43	9.9	
Retired/housewives	44	10.2	
Unemployed	15	3.5	
Other	16	3.7	
**Marital status**			
Never married	166	38.3	
Married	241	55.7	
Widow	18	4.2	
Divorced	7	1.6	
Separated	1	0.2	
**Self-rated health status**			
Poor	26	6.0	
Fair	172	39.7	
Good	142	32.8	
Very good	82	18.9	
Excellent	11	2.5	
**Physical activity**			
Low^a^	302	69.7	
High^b^	131	30.3	
**Smoking**			
Yes	50	11.5	
No	383	88.5	
**Drinking**			
Yes^c^	227	52.4	
No^d^	206	47.6	
**Monthly household income(HKD)**			
<10,000	15	3.5	
10,000–14,999	27	6.2	
15,000–19,999	24	5.5	
20,000–29,999	60	13.9	
30,000–39,999	128	29.6	
≥40,000	179	41.3	

a Five days or over, at least 60 mins vigorous or moderate activities or walking;

b Not meeting the criteria for the “High” group;

c Consuming alcoholic drinks during the last year;

d Consuming zero alcoholic drink during the last year.

### Reliability

According to the corrected item-total correlations (see Table 2), one item with low inter-item correlations (i.e., q18, item-total correlation < 0.2) was deleted. Table 4 summarizes the means, SD, and internal consistency for the scale and subscales without q18. Cronbach's alpha of the total score scale was 0.89, which is satisfactory. The Cronbach's alpha of the subscales ranged from 0.79 to 0.90. The internal consistencies of all subscales are satisfactory. Finally, the scale was composed of 27 items.

**Table 4:** Internal consistency of the HLS-HK (27items).

**Domain**	**No. of items**	**Range of scores**	**Mean**	**SD**	**Cronbach's alpha**
FHL	4	4–20	13.49	3.17	0.84
IHL	7	8–35	24.79	5.15	0.90
CHL-1	6	9–30	20.30	3.93	0.84
CHL-2	5	7–25	20.78	2.80	0.80
CHL-3	5	7–25	14.82	3.55	0.79
HLS-HK	27	51–129	94.18	12.19	0.89

### Validity

The dataset was randomly split into two subsets: for the EFA (*n* = 216), and the other for the CFA (*n* = 217). The sample size for each subsample satisfied the requirement for the sample size, which is larger than 5 times the number of variables for EFA (72), and at least 200 cases for CFA (73).

**Figure 1:**
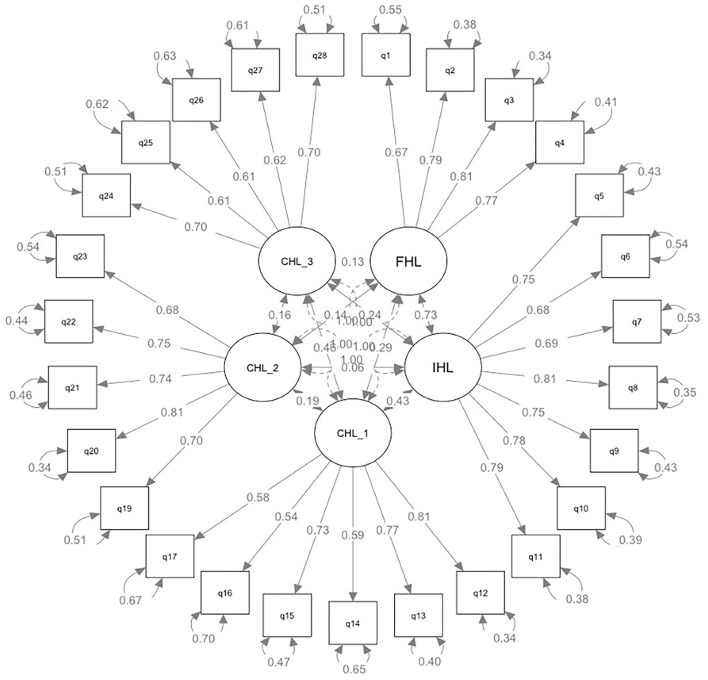
Path diagrams of the CFA model (27 items).

The KMO test showed a score of 0.86, which is above the required 0.70 for conducting EFA. Bartlett's test of sphericity was significant (chi-square = 2,772.356, *p* < 0.000). Therefore, our dataset is suitable for EFA. Regarding the results of EFA, the parallel analysis and scree plot examination suggested five factors with eigenvalues (7.36, 3.15, 2.78, 1.84, and 1.39, respectively) >1, accounting for 53% of the variance (14, 10, 10, 10, and 9%, respectively). Table 5 presents the factor structure of HLS-HK. The four items of FHL all loaded onto the third factor, and seven items of IHL loaded on the first factor. Among the items of CHL, six items of CHL-1 loaded on the second and five items of CHL-2 loaded on the fifth factor, and five items of CHL-3 loaded on the fourth factor. The CFA analysis revealed an acceptable fit of the five-factor model (see Figure 1), with a CFI = 0.91, SRMR =0.06, and RMSEA = 0.06.

**Table 5:** Item scores and factor loadings of HLS-HK (27 items).

**Domain**	**Items**	**Factor loading**				
		**Factor 1**	**Factor 2**	**Factor 3**	**Factor 4**	**Factor 5**
FHL	q1	0.12	0.07	**0.61**	−0.21	0.05
	q2	−0.08	0.02	**0.93**	0.07	−0.04
	q3	0.12	−0.03	**0.73**	−0.03	0.06
	q4	0.14	0.03	**0.61**	0.00	0.03
IHL	q5	**0.45**	0.04	0.31	0.06	0.05
	q6	**0.48**	0.03	0.32	0.05	−0.01
	q7	**0.71**	−0.08	0.09	−0.05	0.05
	q8	**0.85**	−0.03	−0.07	0.00	0.04
	q9	**0.66**	0.09	−0.02	0.10	−0.08
	q10	**0.71**	0.09	0.04	−0.01	−0.03
	q11	**0.74**	0.08	0.02	0.00	−0.01
CHL-1	q12	−0.02	**0.77**	0.07	0.04	−0.04
	q13	−0.02	**0.87**	−0.02	−0.11	−0.01
	q14	0.16	**0.47**	0.01	0.15	0.07
	q15	0.14	**0.66**	−0.06	0.08	0.04
	q16	0.01	**0.46**	0.16	0.11	−0.08
	q17	0.04	**0.48**	0.03	0.17	0.06
CHL-2	q19	−0.10	0.06	0.08	−0.07	**0.66**
	q20	0.08	−0.06	−0.04	−0.01	**0.74**
	q21	−0.04	0.06	0.07	−0.01	**0.60**
	q22	0.01	−0.07	−0.04	−0.03	**0.69**
	q23	0.02	0.01	−0.02	0.11	**0.61**
CHL-3	q24	0.05	−0.08	0.04	**0.80**	−0.14
	q25	0.02	−0.03	−0.02	**0.78**	−0.08
	q26	0.00	0.11	0.13	**0.53**	0.29
	q27	−0.07	0.08	−0.06	**0.65**	0.20
	q28	0.02	0.19	−0.02	**0.62**	0.07

Factor loading in bold are over than 0.40.

### Bivariate analysis

The bivariate analysis showed a strong correlation between the HLS-HK and the HL-SF12 (*r* = 0.67, *p* < 0.001). All subscales of the two scales were significantly associated with each other (Table 6). Compared with FHL (*r*: 0.35–0.46, *p* = 0.000) and IHL (*r*: 0.51–0.64, *p* = 0.000), the three subdomains of CHL (*r*: 0.12–0.35, *p* = 0.000) had lower correlations with the subscales of HL-SF12. The scores on the HLS-HK were significantly associated with education level (H = 7.292, *p* < 0.05), as well as self-rated health status (H = 32.292, *p* < 0.001). However, there was no association between HLS-HK scores and age, income, and health behaviors (Table 7). We further examined the association between these selected variables and scores on the subscales of HLS-HK. There were statistically significant differences in physical activity groups' scores of IHL and age groups' scores of CHL-1 (Table 7).

**Table 6:** Bivariate relationships of HLS-HK scores and HL-SF12.

**Variables**	**HL-SF12**		**FHL**		**IHL**		**CHL-1**		**CHL-2**		**CHL-3**	
	***r***	***p***	***r***	***p***	***r***	***p***	***r***	***p***	***r***	***p***	***r***	***p***
HL-SF12	0.67	<0.001^*^	0.47	0.000^*^	0.68	0.000^*^	0.37	0.000^*^	0.14	0.003^*^	0.38	0.000^*^
Health care	0.63	0.000^*^	0.46	0.000^*^	0.64	0.000^*^	0.35	0.000^*^	0.12	0.013^*^	0.32	0.000^*^
Disease prevention	0.61	0.000^*^	0.41	0.000^*^	0.62	0.000^*^	0.33	0.000^*^	0.12	0.016^*^	0.35	0.000^*^
Health promotion	0.53	0.000^*^	0.35	0.000^*^	0.51	0.000^*^	0.30	0.000^*^	0.14	0.003^*^	0.33	0.000^*^

* p < 0.005.

**Table 7:** Bivariate relationships of HLS-HK scores and other variables.

**Variables**	**HLS-HK**		**FHL**		**IHL**		**CHL-1**		**CHL-2**		**CHL-3**	
	**Mean**	***p***	**Mean**	***p***	**Mean**	***p***	**Mean**	***p***	**Mean**	***p***	**Mean**	***p***
**Age**												
18–54	94.8	0.052	13.5	0.850	24.9	0.590	20.6	0.010^*^	20.7	0.680	15.0	0.251
≥55	92.9		13.4		24.5		19.7		20.9		14.5	
**Education attainment**												
Secondary and below	92.0	0.021^*^	13.4	0.652	24.4	0.430	19.4	0.001^*^	20.7	0.631	14.1	0.004^*^
Post-secondary	95.1		13.6		25.0		20.7		20.8		15.1	
**Monthly household income**												
<10,000	90.1	0.364	12.7	0.121	23.8	0.066	19.3	0.650	20.6	0.273	13.6	0.363
10,000–39,999	94.9		13.8		25.4		20.2		20.6		15.0	
≥40,000	93.5		13.2		24.0		20.5		21.1		14.7	
**Self-rated health**												
Poor	86.9	0.000^*^	11.9	0.016^*^	20.7	0.000^*^	20.0	0.214	21.2	0.031^*^	13.2	0.000^*^
Fair	92.2		13.3		23.8		19.9		21.2		14.1	
Good	94.6		13.8		25.3		20.3		20.5		14.6	
Very good	98.3		13.7		27.0		20.9		20.1		16.7	
Excellent	105.8		15.1		27.6		22.5		21.8		18.9	
**Physical activities**												
Low^a^	94.3	0.551	13.6	0.745	25.1	0.049^*^	20.3	0.774	20.7	0.574	14.7	0.187
High^b^	93.8		13.4		24.1		20.2		21.0		15.2	
**Smoking**												
Yes	93.1	0.508	12.8	0.134	24.5	0.659	20.0	0.303	20.9	0.800	14.9	0.859
No	94.3		13.6		24.8		20.4		20.8		14.8	
**Drinking**												
Yes^c^	94.6	0.324	13.6	0.776	25.1	0.193	20.3	0.885	20.8	0.995	14.9	0.707
No^d^	93.8		13.4		24.5		20.3		20.8		14.8	

* p < 0.05;

a Five days or over, at least 60 mins vigorous or moderate activities or walking;

b Not meeting the criteria for the “High” group;

c Consuming alcoholic drinks during the last year;

d consuming zero alcoholic drink during the last year.

## Discussion

We proposed a scale within 27 items encompassing a range of HL competencies and addressed the shortage of HL measurement in Hong Kong. In the scale development process, we generated the original items by systematically searching the published literature relevant to the construct of HL to cover its full breadth and depth. We invited healthcare users and providers to examine the newly developed scale's content validity and face validation. The scales' two novel aspects related to Nutbeam's framework are discussed in the below section.

### Scale novelty

First, compared with previous scales (14, 30–35), this scale fully operationalized the three domains identified by Nutbeam. In the domain of FHL, we formulated five items to examine subjects' abilities to read and understand health-related information in clinical and non-clinical settings. To measure IHL, seven items were generated to examine the abilities that people need to gain health-related information in daily life as well as healthcare consulting. Regarding CHL, this scale is more explicitly linked to the latest understanding of this domain. As introduced, scholars advocated that CHL is more than the ability to analyze health-related information critically and should reflect societal influences on health knowledge, beliefs, and behaviors (26–28, 74, 75). Therefore, we built a set of items to thoroughly measure this domain and divided them into three categories. Specifically, in CHL-1, we drew items from existing research to examine subjects' skills to judge the quality of information; In CHL-2, we selected the most fundamental and non-medical causes of individuals' lifestyles from the WHO report to test people's knowledge of SDH (76); In CHL-3, we examined people's activities to address SDH at the social level and interpersonal level.

Second, this scale provides new insights into the domain of CHL in Nutbeam's framework, as it was developed in non-western countries. Previous studies (74, 77–79) emphasized the importance of collective action to address social determinants of health to measure CHL. They argued that individuals with sufficient CHL skills tended to participate in social and political movements for change, including informed voting and advocacy for health issues. However, we proposed that the action should not be narrowed to this social level. This is because solely focusing on participation in collective action may not fully capture the CHL level of certain population groups with limited resources to participate in political action to shape a better society, such as Hong Kong people. In fact, emerging studies (28, 80–84) recognized the importance of interpersonal-level activities to address social determinants of health. It can be assumed that people with sufficient CHL skills may transfer the knowledge of social structural factors of health into actions to get peer support or build a supportive environment for health at the interpersonal level. For instance, one newly developed scale focusing on adolescents' CHL also included individuals' ability to provide social support and participate in democratic actions about health as its component (84). In HLS-HK, we formulated three questions (i.e., q26-q28) to complement the measurement of interpersonal-level actions to address social determinants of health and, in turn, increase this domain's cultural sensitivity across different cultures.

### Scale validation

Overall, the scale is reliable and valid. The internal consistency for the scale and subscales are satisfactory. EFA produced a five-factor solution, and CFA revealed an acceptable fit of the five-factor model. These results confirmed that the 27-item of HLS-HK represents the framework as initially designed. The convergent validity of HLS-HK was achieved by using HL-SF12 as the reference tool. Further, we compared the correlation between the subscales from the two scales. The three domains under the CHL level rather than FHL or IHL, had weak relationships with the subscales of HL-SF12. This may be because our scale measured several HL skills missed in HL-SF12. This result supports the research gap mentioned at the beginning: limited operationalization of CHL in previous scales.

The q18 was excluded because of its low item-total correlation. Our previous Delphi study already showed divergent opinions on this item among health professionals and laypeople. Although laypeople acknowledged the influence of socioeconomic factors on health, they tended to believe personal lifestyle substantially affects health. In contrast, health professionals can fully understand the effect of social structural factors on health, which can be more important than lifestyle choices at some point. Regarding this divergence, previous studies highlighted that laypeople might be more likely to understand and express the idea about social disadvantage and health through a contextualized narrative description of their own experiences compared with answering fixed choice questions (74). Hence, it is not surprising that we did not get concise answers on this item, which caused its low correlation with other items.

This study showed that HLS-HK is significantly associated with education. It was possible to observe a higher proportion of individuals with a high level of education (i.e., post-secondary) among those with a better score on HLS-HK. This finding is similar to studies elsewhere (85, 86). There are two potential explanations for this finding. First, highly educated groups have access to information and resources needed for better health outcomes, while low-educated groups often lack these resources. Second, advanced education usually provides a higher level of cognitive skills to process and use information compared with primary education. For instance, in Hong Kong, health promotion programmes under the Healthy School Policy in primary and secondary schools mainly cover basic health knowledge (87–89). Contrarily, health education among local university students focuses on problem-solving skills for a range of health issues (90, 91), which are beneficial for them to develop sophisticated HL skills. Hence, it is crucial to set up systems for universal access to health-related sources and create easy-to-understand health education materials for the general public. Moreover, HL is dynamic, not unchangeable. It can be improved by providing information, effective communication, and structured health education programs. In this respect, HL is a critical concept for reducing health inequalities.

Besides, a positive association between HLS-HK and self-rated health was obtained. This result is consistent with previous studies (9, 92, 93). However, there is no association between the whole scores of HL and health behaviors, age, and income in our study. It is still unclear how health literacy competencies might contribute to individual or community health outcomes and how such competencies might be affected by social status factors. Several conceptual models of the pathways linking HL to health outcomes have been proposed (94, 95). As noted in the models, the paths among antecedent factors (e.g., income and age), HL, and health-related behaviors and outcomes are complicated (94). Health system-level moderators (e.g., healthcare system and healthcare providers) and societal-level moderators (e.g., culture, community resources, and family) can affect the pathway between HL and health outcomes (94). In the present study, the association between these health-related variables and scores on the subscales of HL provided more details on the paths. For instance, the negative association between CHL-1 and age was evidenced in the study. It seems reasonable that critically analyzing information is more difficult for older individuals because of age-related cognitive decline. To summarize, comprehensive knowledge of HL in the general population is essential to guide health systems and organizations to achieve better health outcomes. More empirical studies are warranted to better understand the pathways and guide effective health promotion policies and programs in the future.

### Study limitations

Several limitations of this study should be noted. First, selection biases might exist. The study subjects were recruited from an online questionnaire platform registers who may be better at seeking and understanding information and are interested in health issues. It is not surprising that in the present dataset, most participants were highly educated or have high incomes who may have more resources to access and use information, and healthy people who may often use the Internet to search for healthy lifestyle advice. Moreover, because we set up an attention check question, older adults who were more easily distracted may have failed to return a complete form and therefore were excluded from the analysis. Further study is needed to examine whether the HLS-HK is acceptable to people with a wide range of HL levels. Second, respondents may overestimate their HL using a self-reported scale. Due to resource limitations, the present study did not use a performance-based measure as the comparison scale, so the overestimation effect needs to be further explored. Third, it might be possible that some aspects of CHL were not included in the measure due to the complexity of this domain. Although we used a deductive approach *via* literature review to generate items, more diverse views may be considered to reflect more comprehensive perspectives of this domain. For example, further research to study residents' insights about transferring knowledge into action to address social determinants of health *via* focus groups may be needed.

### Implications for policy and practice

Despite its limitations, the implication of this study should be highlighted. First, this scale HLS-HK can be used to comprehensively measure FHL, IHL, and CHL. The true promise of one HL scale is not to simply screen people according to their HL level but also should be to inform and tailor future interventions to enhance their HL. Therefore, using a comprehensive HL measurement such as HLS-HK can benefit healthcare workers, policymakers, and researchers to better understand the difficulties and barriers that service users may encounter when they use health-related information and services (e.g., found difficulty in understanding medical jargon, embarrassed to ask questions during medical consultation, overwhelmed by information overload, didn't realize the societal benefit of vaccination, and low interests to take action for public health) and further design interventions to address these issues. Next, a further possible use of HLS-HK is as a tool to explore the path between HL and health outcomes. A clear link between HL and health outcomes could result in higher quality and more effective interventions. As noted, more empirical studies (e.g., cross-sectional survey and longitudinal study) are needed to examine the impacts of HL on healthcare, such as how HL leads to healthy behaviors across different age groups and different utilization rates of screening programmes by place of residence in Hong Kong. Finally, HL is an evolving construct, and there is no consensus on its components. We hope our work could contribute to a more comprehensive understanding of HL as a social construct rather than a set of skills related to information transmission. Of course, the scale needs to undergo rigorous testing with diverse population groups so that it can be used to evaluate and compare HL across cultures. Other researchers can use or amend this scale for their research interests and target populations' needs. For example, scholars in a democratic country can use this scale to learn how citizens transfer their understanding of health into actions for personal health and community health in depth. For scholars in an autocratic society, however, they may need to contextualize the items of CHL as residents might have low motivation and resources for social participation for health.

## Conclusion

The HLS-HK is valid and reliable for evaluating HL in Hong Kong. This scale can measure FHL, IHL, and CHL in clinical and public health contexts. It also extended the operationalization of the above domains and fully operationalized the CHL *via* three subdomains. HLS-HK, with a testable framework and multifaceted attributes, will be validated in more countries and populations to advance this field of science further.

## Data availability statement

The raw data supporting the conclusions of this article will be made available by the authors, without undue reservation.

## Ethics statement

The studies involving human participants were reviewed and approved by Survey and Behavioral Research Ethics Committee of the Chinese University of Hong Kong. The patients/participants provided their written informed consent to participate in this study.

## Author contributions

EW, PM, DD, and CT designed the study. CT collected and analyzed the data, wrote the draft manuscript, and critically revised the manuscript. EW was responsible for data curation and project administration. DD and PM were incharge of project administration and supervision. AC assisted with the funding acquisition and project administration. EW, PM, and DD commented and edited the whole draft. All authors read and approved the final manuscript.
