# Supplementary material for: Development and validation of a comprehensive health literacy tool for adults in Hong Kong
Source: Front Public Health. 2023 Jan 10;10:1043197. doi: 10.3389/fpubh.2022.1043197 (PMC9871493; doi:10.3389/fpubh.2022.1043197)
Supplement: Supplementary file 1 [file Table_1.docx]

**Appendix 1** Results of cognitive interviews

| **Domain** | **Items** | **Wording changes after interviews** |
| --- | --- | --- |
| FHL | How often do you: |  |
|  | …need help when you are given information to read by your doctor, nurse or pharmacist | Deleted ^[1]^ |
|  | …need help when you fill out medical forms by your doctor, nurse or pharmacist | Changed to “ … need help when you fill out medical forms” ^[2]^ |
|  | ...find that characters cannot understand when you read instructions or leaflets from hospitals or clinics | No change |
|  | …feel that the content is too difficult to understand when you read instructions or leaflets from hospitals or clinics | No change |
|  | ... have problems learning about your medical condition because of difficulty understanding health-related written information | Changed to “ …have problems understanding health-related written information” ^[3]^ |
| IHL | How easy would you say it is to: |  |
|  | …find related information when you are ill and have questions on disease or health problems | No change |
|  | ...find related information when you are not ill but want to do something to further improve your health | No change |
|  | …give all the information a doctor, nurse, or pharmacist need when you talk to them | Changed to  “When you talk to a doctor, nurse, or pharmacist, how difficult would you say it is to:  …give all the information they need |
|  | …ask the questions you want to ask when you talk to a doctor, nurse, or pharmacist | …ask the questions you want to ask |
|  | …extract the information you want when you talk to a doctor, nurse, or pharmacist | …ask further explain anything that you do not understand after they answer your questions |
|  | … ask a doctor, nurse, or pharmacist explain anything that you do not understand | …extract the information you want |
|  | …understand the obtained information when you talk to a doctor, nurse, or pharmacist | …understand the obtained information” ^[4]^ |

**Appendix 1** (continued)

| **Domain** | **Items** | **Wording changes after interviews** |
| --- | --- | --- |
| CHL-1 | When you get information for health in daily life, how often do you consider the following: |  |
|  | …whether the information source is credible | No change |
|  | …whether the information content is valid and reliable | No change |
|  | …whether the publish time is appropriate | No change |
|  | …whether other reliable sources support the facts or conclusions of this source | No change |
|  | …whether the person or organization that produced the information have a bias | No change |
|  | …whether the information is applicable to you | No change |
| CHL-2 | How do you agree about the following ^d^: |  |
|  | …socioeconomic status affects health | No change |
|  | …stress affects health | No change |
|  | …being isolated from the community and workplace impacts health | No change |
|  | …having little control over one’s work impacts health | No change |
|  | …poor childhood experience has an impact on one's physical/mental health when he or she becomes an adult | No change |
|  | …good social relations contribute to health | No change |
|  | …transportations impacts health | Deleted ^[5]^ |
| CHL-3 | How often do you:  …participate in government’s programmes about health promotion and disease prevention | Changed to “…promote government to launch programmes about health promotion and disease prevention” ^[6]^ |
|  | …participate in community’s initiatives in health promotion and disease prevention | Combined the two items as “…participate in community’s or non-governmental organisations’ initiatives in health promotion and disease prevention” ^[7]^ |
|  | …participate in non-governmental organisations’ initiatives in health promotion and disease prevention |  |
|  | …help your family members or a friend when they had questions concerning health issues | No change |
|  | …seek information from others when you come up with questions concerning a health issue | No change |
|  | …share and communicate your opinion about illness when you talk to a family member or friend | No change |

Notes:

[1] = Reason: interviewees commented that the scenario did not frequently occur in hospital or clinic;

[2] = Reason: interviews suggested that the item was wordy;

[3] = Reason: interviewees commented that this item was too wordy;

[4] = Reason: interviewees complained that these sentences are too long, and “when you talk to a doctor, nurse, or pharmacist” repeated.

[5] = Reason: over half of the participants felt confused about the meaning of the item. They did not understand why transportation impacts health;

[6] = Reason: interviewees asked what’s the difference between government’s programmes and community’s programmes about health promotion and disease prevention;

[7] = Reason: interviewees could not differentiate between the “community’s initiatives” and “non-governmental organizations’ initiatives.
